# Imperfect Vaccine Aggravates the Long-Standing Dilemma of Voluntary Vaccination

**DOI:** 10.1371/journal.pone.0020577

**Published:** 2011-06-08

**Authors:** Bin Wu, Feng Fu, Long Wang

**Affiliations:** 1 State Key Laboratory for Turbulence and Complex Systems, Center for Systems and Control, College of Engineering, Peking University, Beijing, China; 2 Program for Evolutionary Dynamics, Harvard University, Cambridge, Massachusetts, United States of America; University of Maribor, Slovenia

## Abstract

Achieving widespread population immunity by voluntary vaccination poses a major challenge for public health administration and practice. The situation is complicated even more by imperfect vaccines. How the vaccine efficacy affects individuals' vaccination behavior has yet to be fully answered. To address this issue, we combine a simple yet effective game theoretic model of vaccination behavior with an epidemiological process. Our analysis shows that, in a population of self-interested individuals, there exists an overshooting of vaccine uptake levels as the effectiveness of vaccination increases. Moreover, when the basic reproductive number, 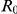, exceeds a certain threshold, all individuals opt for vaccination for an intermediate region of vaccine efficacy. We further show that increasing effectiveness of vaccination always increases the number of effectively vaccinated individuals and therefore attenuates the epidemic strain. The results suggest that ‘number is traded for efficiency’: although increases in vaccination effectiveness lead to uptake drops due to free-riding effects, the impact of the epidemic can be better mitigated.

## Introduction

Preemptive vaccination is the principle strategy for the intervention and control of infectious diseases. However, vaccination represents a long-standing social dilemma for public health administration. On the one hand, compulsory vaccination may result in an infringement of civil rights [1]. On the other hand, voluntary vaccination cannot lead to sufficiently high herd immunity for disease eradication. Thus it often fails to protect populations from epidemics [2], [3], [4], [5].

Traditional epidemiological modeling focuses on the pathway of disease transmission, and often does not take into account human strategic behavior in response to the epidemic [6]. However, it is more plausible to integrate human behavior with the epidemiological process. In this sense, voluntary vaccination itself is a social dilemma: vaccinated individuals can escape from the disease with a cost partly incurred by the vaccine side effects; the unvaccinated can also be protected from the epidemics without paying anything provided the population immunity is in effect. In this case, self-interested individuals attempt to shun vaccination while still benefitting from the herd immunity. Such free-riding may lead to a low vaccination level, failing to eradicate the disease, thus a social dilemma [7], [8]. The framework of game theory properly describes how individuals react when facing a dilemma [9], [10], [11], [12], [13], [14], [15], [16], [17], [18], [19]. In particular, how the evolutionary outcome of the social dilemma is achieved can be investigated based on the imitation process [20], [21]. Therefore, voluntary vaccination can be studied in this framework and noteworthy there has been an emerging literature of combining epidemiology and game theory [7], [22], [23], [24], [5], [25], [8], [26].

Previous work usually assumes perfect vaccination, i.e., the vaccinated individuals gain perfect immunity against the disease [7], [23], [8]. The effectiveness of vaccination, however, is not 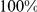, such as measles [27], malaria [28] and HIV [29]. Even though the actual vaccination is perfect, the perceived effectiveness can be not. Questionnaire results have shown the perceived effectiveness is often lower than the actual one [24]. This perceived efficacy of vaccination, influenced by psychological effects, plays a determinant role since individuals adjust their strategic behavior based on perceptions of the vaccine efficacy rather than the actual one [5], [24]. Therefore, imperfect vaccination should be taken into account in the game theoretical analysis of the vaccination behavior [30], [31], [32]. Besides, public concern towards the effectiveness of vaccine is so common that it often leads to massive vaccine avoidance. How vaccine effectiveness affects vaccination level and thus the severity of epidemic outbreak has not yet been fully answered. Motivated by these, we study this problem by a minimal model.

## Analysis

For proof of principle, we consider vaccination dynamics in an infinitely large well mixed population. In addition, we assume that individuals have a perfect knowledge on the effectiveness of the vaccination. In this case, there is only one parameter describing both the actual and the perceived effectiveness.

The vaccination game consists of two stages, the yearly vaccination campaign and an epidemic season. During the vaccination campaign, each individual decides whether or not to take vaccination. A vaccinated individual pays a cost 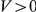 while an unvaccinated individual pays nothing. This cost 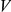 includes the time spent in taking the vaccination as well as its side effects. During the epidemic season, the population can be divided into two parts: one comprises effectively vaccinated individuals, and the rest is composed of unvaccinated individuals and the vaccinated ones whose vaccinations are not effective. Successfully vaccinated individuals are immune to the seasonal disease, and thus have no risk of getting infected. For the remaining individuals, however, they become infected with a probability 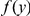, where 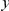 is the frequency of effectively vaccinated individuals. In this case the infected bear a cost by 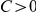. This cost 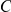 includes expenses and time for health care as well as mortality. The larger the number of effectively vaccinated individuals is, the less likely an unvaccinated individual gets infected. Thus 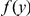 is decreasing with 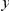.

Let the effectiveness of the vaccination be 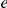 and the vaccine uptake level be 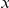. The frequency of the effectively vaccinated individuals is 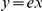. The fraction of the vaccinated and healthy individuals is 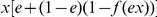, which is composed of two parts: these effectively vaccinated individuals (with frequency 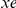) and those ineffectively vaccinated individuals (with frequency 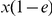) who are free from the infection (with frequency 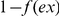). In this case, each effectively vaccinated individual gets payoff 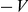. In analogy to this, the frequencies and payoffs for different individuals are given by Table 1.

**Table 1:** The fraction and the payoff for the four types of individuals in the population.

	Vaccinated & Healthy	Vaccinated & Infected	Unvaccinated & Healthy	Unvaccinated & Infected
Fraction	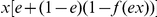	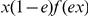	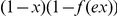	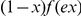
Payoff	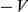	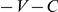	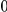	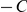

They are the vaccinated and healthy, the unvaccinated and healthy, the vaccinated and infected and the unvaccinated and infected.

When the epidemic season ends, i.e., the average abundance of infected individuals does not change, individuals adjust their strategies by imitation where successful individual's strategy is more likely to be followed [33], [34]. Here we employ the Fermi update rule to characterize such an imitation process [35], [36], [8], [37], [38]: two individuals 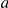 and 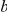 are selected randomly; 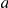 learns to behave like 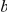 with probability
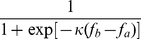
(1)


where 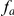 and 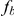 are the perceived payoffs for 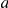 and 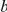, and 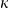 is the selection intensity indicating how strongly individuals are responsive to payoff difference.

The dynamics of the vaccination is governed by [20], [39]

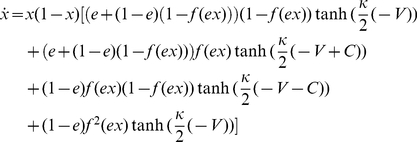
(2)


It has been suggested that the selection intensity for human imitation is rather weak [21], [34], i.e. 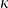 is sufficiently small. We perform the Taylor expansion of the r.h.s of Eq. (2) in the vicinity of 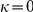, then after a time rescaling which does not change the dynamics, Eq. (2) can be captured by a much more simple form
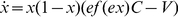
(3)


In what follows, we investigate how the vaccine uptake evolves by Eq. (3) for general function of infection risk 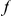. To this end, we focus on how the effectiveness of vaccination has an impact on the the collective outcome of vaccination behavior and the effective vaccination level. Then we incorporate an epidemic dynamics to obtain a specific infection function. Based on this, we provide precise predictions for the two problems. Besides we also study how the effectiveness affects the final epidemic size in this case.

### General infection function

For a general function of infection risk 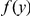, when 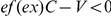 is valid for all 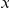 lying between zero and one, no one would take vaccination in the long run, i.e. 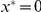 is the unique stable equilibrium for Eq. (3). Since 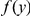 is a decreasing function, 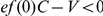 is sufficient to ensure 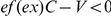. In analogy to this, when 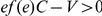 is valid, the entire population ends up with full vaccination, i.e. 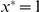 is the unique stable equilibrium. For 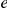 fulfilling 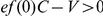 and 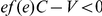, by the monotonicity of 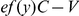 in 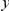, there is a unique internal equilibrium,
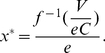
(4)


Further, 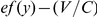 is decreasing, the derivative at 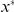, namely 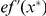, is negative. Thus 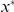 is stable, indicating the coexistence of the vaccinated and the unvaccinated. To show how 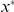 is affected by 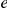 requires the exact form of the function of infection risk. We will address it later.

The effective level of vaccination reads
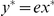


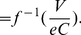
(5)


By Eq. (5), 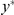 is an increasing function of the effectiveness, 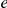. In other words, the effectively vaccinated level always increases with vaccine efficacy. This result only requires that 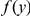 decreases with 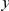. This is true for most, if not all, known infection functions [22], [23]. Therefore our predictions are robust with respect to variations in specific infection functions.

### A specific infection function

In order to give precise predictions, we adopt a simple Susceptible-Infected-Recovered (SIR) model with demographical effects as presented in [7]. In this model, the population is divided into three different compartments: susceptible, who are healthy but can catch the disease if exposed to infected individuals; Infective, who are infected and can pass the disease on to others; Recovered, who are recovered from the infection and gain immunity against the disease. The time evolution of the population states is governed by the following equations
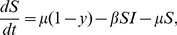
(6)

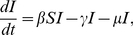
(7)

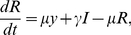
(8)where 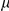 is the birth rate and equal to the mortality rate (for simplicity, we only consider constant population size), 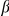 is the transmission rate, 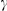 is the recovery rate, and 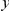 is the fraction of effectively vaccinated individuals among newborns.

From Eq. (7), we derive the basic reproduction ratio 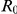: if 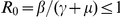, the time derivative of 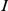 is negative, suggesting that the disease cannot persist in the population. The equilibrium state of the population consists of 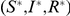, with 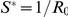, 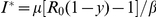 and 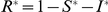. By setting 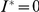, we obtain the herd immunity needed to eradicate the disease, 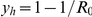.

Based on this stationary equilibrium, we calculate the probability that an unvaccinated individual gets infected in her life time. The waiting time to acquire infection follows an exponential distributions with rate 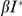, and so does the waiting time to death but with rate 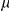. Since infection and death are two independent processes, the probability that infection occurs before death event is the relative ratio of intensities, 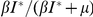. This probability gives the infection risk of an unvaccinated individual, namely, 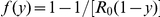 which is a function of the population level of effective vaccine uptake 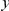 and holds for 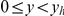. When 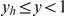, 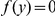, i.e. the disease will be eradicated provided the effective level of vaccination exceeds the critical point 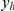. Thus we have
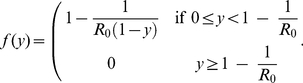
(9)


**Figure 1:**
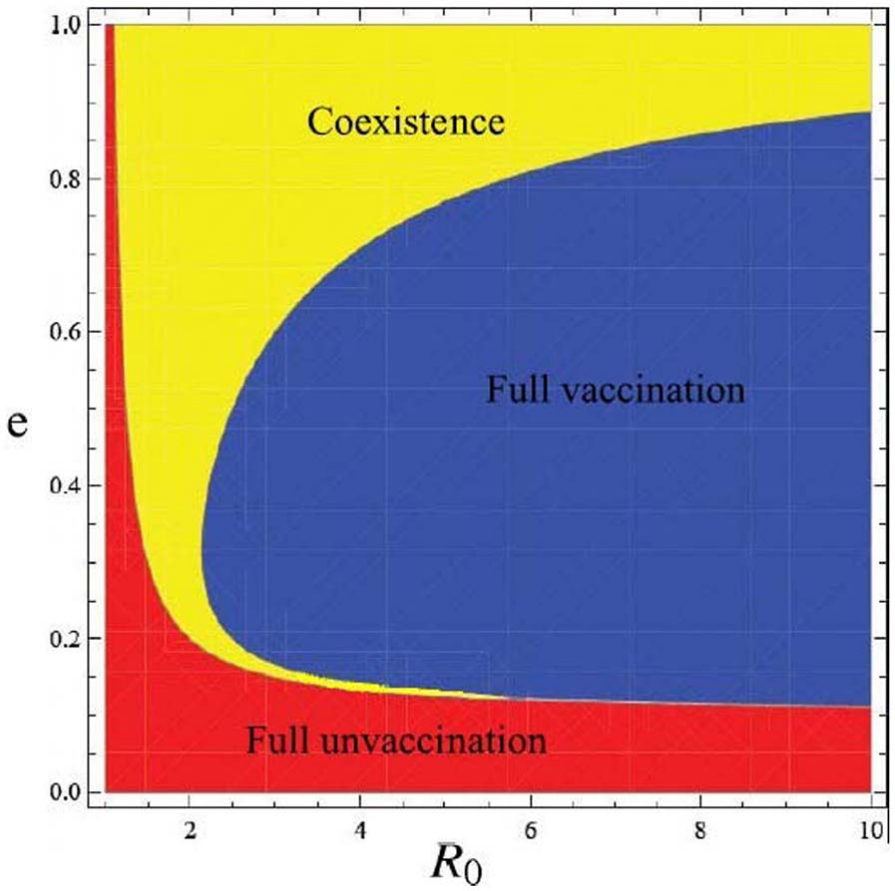
The vaccination behavior on the basic reproductive ratio 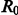 **and the effectiveness** 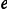. Here 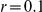, where 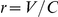. See main text for details.

Taking this specific infection function Eq. (9) into Eq. (3), we present the full dynamics analysis of the evolution of vaccination behavior in the long run (see Fig. 1). Let the ratio of the vaccination cost versus the infection cost 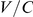 be 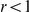. We have (For details, see Text S1)

Case 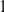: when 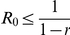, all are unvaccinated for 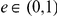.

Case 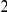: when 
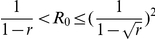
; if 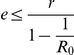, all are unvaccinated, otherwise there is a unique internal stable equilibrium 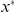.

Case 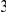: when 
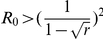
; if 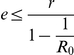, all are unvaccinated, if 
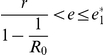
, there is a unique internal stable equilibrium 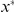, if 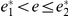, all are vaccinated, if 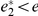, there is a unique internal stable equilibrium 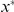.

Where 
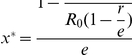
, 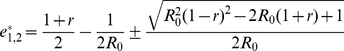.

**Figure 2:**
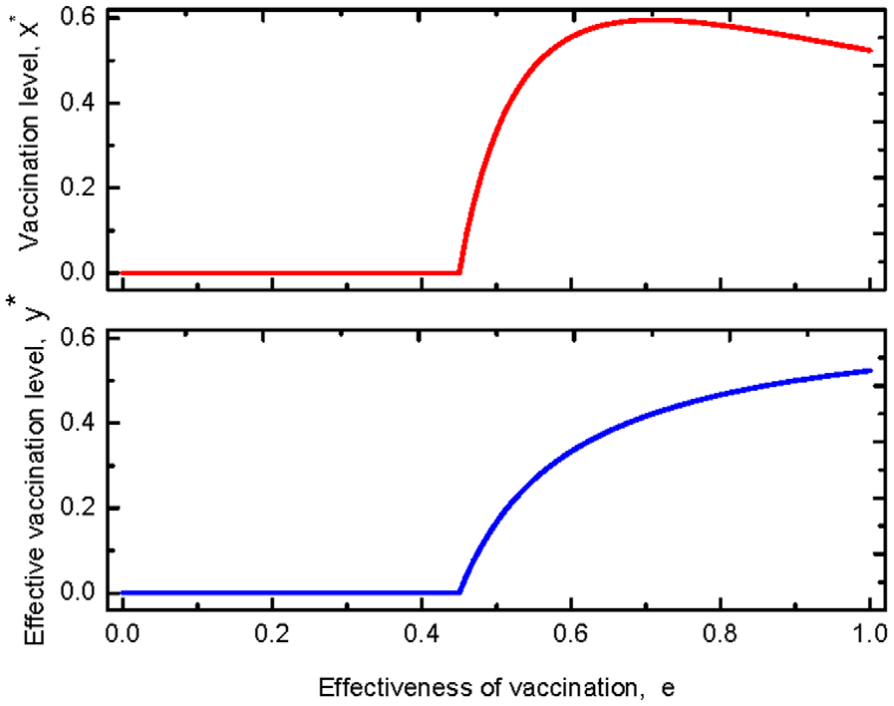
Fractions of the vaccinated and the effective vaccinated for a disease with a moderate infectiveness. The upper panel shows the stationary frequency of the vaccinated with respective to the effectiveness. No one takes vaccination until it is sufficiently efficient, 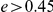. Then the vaccine uptake level increases with the effectiveness. When the effectiveness exceeds a threshold, 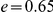, however, the vaccination level decreases with the effectiveness. The lower panel shows the stationary abundance of the effectively vaccinated individuals with respect to the effectiveness. It is shown the efficient vaccinated individual increases with the effectiveness all the time. Thus the behavior of vaccination and the impact of the vaccination against epidemic are not in agreement: for high effectiveness, even though vaccination rate is decreasing, the number of effectively vaccinated individual increases as the effectiveness 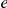 increases. Here 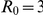, 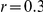 satisfying 
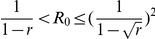
.

**Figure 3:**
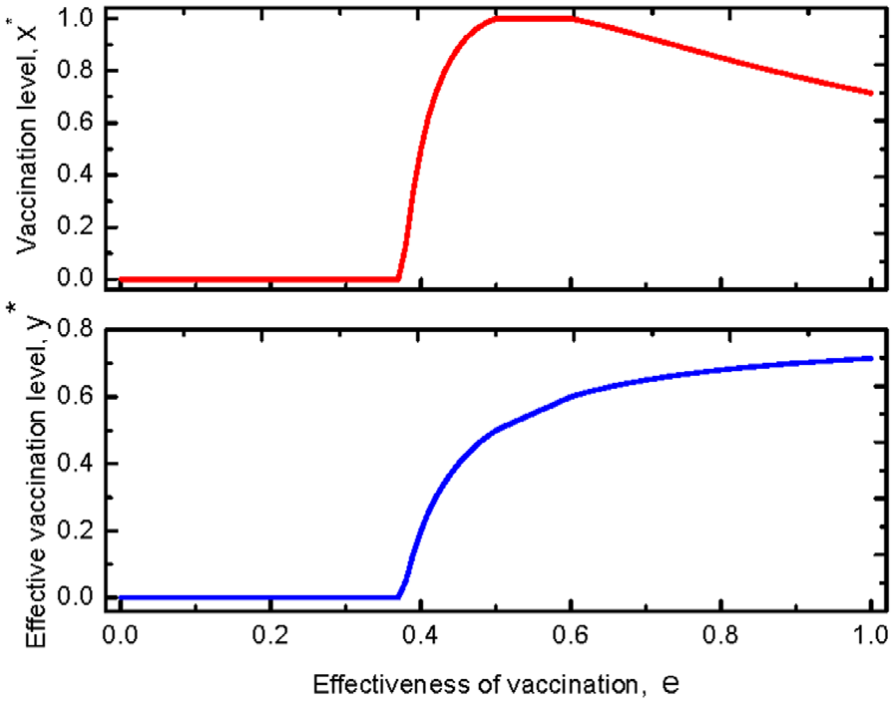
Fractions of the vaccinated and the effective vaccinated for a serious disease. The upper panel shows the stationary frequency of the vaccinated with respective to the effectiveness. Compared to Fig. (2), the whole population could take vaccination provided the effectiveness is moderate, 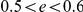. The lower panel indicates the stationary abundance of the effectively vaccinated individuals with respect to the effectiveness. Compared with Fig. (2), the frequency of the effective vaccinated individual also increases with the effectiveness, but it is higher than that of Fig. (2). Here 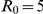, 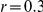 satisfying 
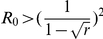
.

Case 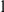 indicates that for a mild epidemic, 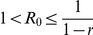, vaccination behavior is impossible for any vaccination effectiveness. For a more serious epidemic, Case 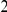 shows, however, there is an overshooting of vaccine uptake: the coexistence of the vaccinated and the unvaccinated emerges as the effectiveness exceeds a threshold. Furthermore, interestingly, the increase in effectiveness does not always promote the vaccination behavior (see the upper panel of Fig. 2). Intuitively, for the vaccinated, increasing the vaccination effectiveness does reduce the infection probability. For the unvaccinated, however, this leads to that they are protected by a even more effective herd immunity. Thus increasing the effectiveness of vaccination is beneficial both to the vaccinated and to the unvaccinated. The two strategies compete with each other and the more beneficial one is more likely to spread through imitation. The result shows, when the effectiveness is below the critical value, the more beneficial one is the vaccinated. When it exceeds the critical value, the more beneficial one is the unvaccinated. Mathematically, the non-monotonicity of 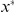 on 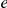 is induced from the non-monotonicity of 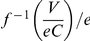 as discussed above. For an even more serious epidemic, Case 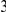, the dynamics of the vaccination behavior is qualitatively identical to that of Case 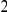. However, in contrast with Case 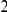, full vaccination can be reached (see the upper panel of Fig. 3).

Besides the vaccination behavior, by taking Eq. (9) into Eq. (5), the effective vaccination frequency, 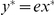 is given by
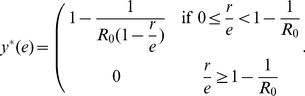
(10)


Hence, the effective vaccination frequency increases as the effectiveness increases as predicted (See the lower panels of Figs. 2 and 3).

**Figure 4:**
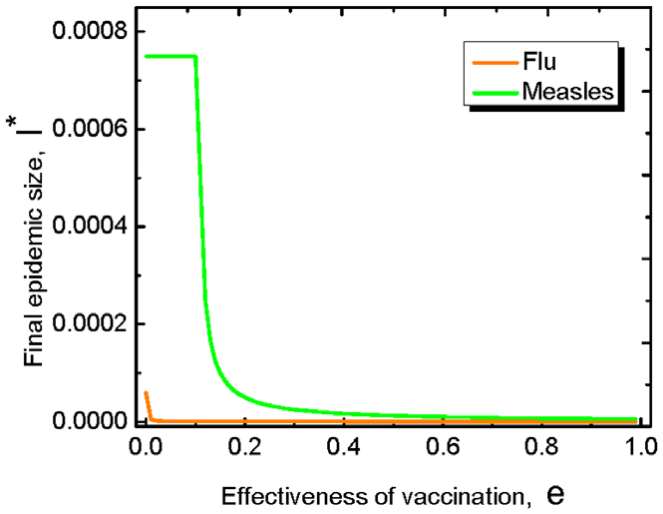
Final epidemic size 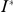 **for the flu and the measles.** The final epidemic size here is the average abundance of the infected individual in the long run. For both the flu and the measles, the final epidemic size decreases with the effectiveness. Here 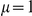 is the birth rate of the population. For the measles, 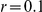, 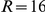 and 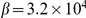; For the flu 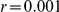, 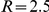 and 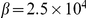 [6], [7].

Further, it is of interest to investigate how the final epidemic size is influenced by the effectiveness of the vaccination. The final epidemic size 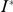 here refers to the average fraction of the infected individuals at the end of the epidemics. For the SIR model with vital dynamics discussed above, when the vaccine uptake reaches a stationary level 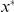, the final epidemics size of the population is given by
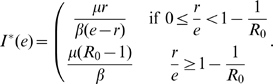
(11)Therefore, 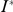 is a decreasing function with 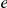. That is to say, the more effective the vaccination is, the smaller proportion is infected eventually. In particular, we find this is true for the flu and the measles (see Fig. 4).

## Discussion

Voluntary vaccination is the principle strategy to control epidemic outbreaks. Vaccination itself, however, is a social dilemma [8]. Evolutionary game theory, which describes the evolution of strategies in self-interested individuals, is a powerful mathematical framework to study such social dilemmas. Most previous works employing this framework are based on the assumption of perfect vaccination, where epidemics can be eradicated from the vaccinated. The vaccination, however, cannot be so effective [27], [28], [29]. Therefore it is of interest to ask how the effectiveness of the vaccination has an impact on the vaccination.

To this end, we combine the SIR model with the imitation dynamics. For the spreading of disease, we find that increasing the effectiveness of vaccination always inhibits the prevalence of epidemics. Therefore imperfect vaccine aggravates the long-standing dilemma of voluntary vaccination. Thus to control the epidemics, i.e. to enhance the vaccination effectiveness, there are two ways: one is to improve technology in vaccine: increasing the actual effectiveness of the vaccination. The other is to make use of media: enhancing the perceived effectiveness.

For the vaccination behavior, we find that when the epidemic is sufficiently serious, all the self-interested individuals may take vaccination for an intermediate vaccine efficacy. In other words, increasing effectiveness inhibits the prevalence of the epidemic with a declining vaccination level. For example when 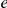 is larger than 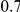 in Fig. 2 and larger than 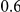 in Fig. 3. This suggests even though the vaccination level decreases with effectiveness sometime, the epidemic is still better controlled than before, thus it is not necessary to be panic. Besides, all the above results are robust to general imitation processes [34].

Here we study the simplest possible case, i.e., well-mixed populations, for proof of principle. A natural extension of the present analysis is to take population structure into account. For instance, we can consider spatial structure, which restricts the neighborhood of individuals whom one can infect or imitate. In doing so, however, the evolutionary dynamics of vaccination behavior become more complex and require separate, in-depth studies. In essence, the vaccination game is similar to the well-studied snowdrift game [40]. Therefore, spatial structure acts as a “double-edged sword” [8]. In particular, spatial structure promotes vaccination behavior for small vaccination costs, and thus we expect that the critical efficacy of vaccination above which vaccination behavior persists should be smaller compared to the well-mixed case. These extensions are promising areas for future research.

## Supporting Information

Text S1 Dynamics analysis.
(PDF)
