# Supplementary material for: Imperfect Vaccine Aggravates the Long-Standing Dilemma of Voluntary Vaccination
Source: PLoS One. 2011 Jun 8;6(6):e20577. doi: 10.1371/journal.pone.0020577 (PMC3110791; doi:10.1371/journal.pone.0020577)
Supplement: Text S1 — Dynamics analysis. (PDF) [file pone.0020577.s001.pdf]

# Text Supplementary 1 - Imperfect vaccine aggravates the long-standing dilemma of voluntary vaccination

Bin Wu[1*], Feng Fu[2], Long Wang[1]

**1 Center for Systems and Control, State Key Laboratory for Turbulence and Complex Systems, College of Engineering, Peking University, Beijing, China**

**2 Program for Evolutionary Dynamics, Harvard University, Cambridge, Massachusetts, USA**

∗ **E-mail: bin.wu@evolbio.mpg.de**

# Dynamics analysis

In the following, we show the dynamics analysis for

$$\dot{x} = x(1-x)(ef(ex)C - V) \tag{1}$$

where the function of infection risk is given by

$$f(x) = \begin{cases} 1 - \frac{1}{R_0(1-x)} & \text{if } 0 \leq x < 1 - \frac{1}{R_0} \\ 0 & x \geq 1 - \frac{1}{R_0} \end{cases} \tag{2}$$

Let $G(x) = ef(ex) - r$, where $r = V/C$. The above replicator equation is equivalent to

$$\dot{x} = x(1-x)G(x) \tag{3}$$

under a time rescaling [1].

**Theorem** For the evolutionary outcome depicted by the replicator equation, we have

$(i)$ When $R_0 \leq \frac{1}{1-r}$; all are unvaccinated.

$(ii)$ When $\frac{1}{1-r} < R_0 \leq (\frac{1}{1-\sqrt{r}})^2$; if $e \leq \frac{r}{1-\frac{1}{R_0}}$, all are unvaccinated, otherwise there is a unique internal stable equilibrium $x^*$.

$(iii)$ When $R_0 > (\frac{1}{1-\sqrt{r}})^2$; if $e \leq \frac{r}{1-\frac{1}{R_0}}$, all are unvaccinated, if $\frac{r}{1-\frac{1}{R_0}} < e \leq e_1^*$, there is a unique internal stable equilibrium $x^*$, if $e_1^* < e \leq e_2^*$, full vaccination, if $e_2^* < e$, there is a unique internal stable equilibrium $x^*$.

Where $x^* = \frac{1-\frac{1}{R_0(1-\frac{r}{e})}}{e}$, $e_{1,2}^* = \frac{1+r}{2} - \frac{1}{2R_0} \pm \frac{\sqrt{R_0^2(1-r)^2 - 2R_0(1+r)+1}}{2R_0}$.

*proof*

For $(i)$:

since $f(y)$ is decreasing, thus $G(x)$ is decreasing. Therefore if $G(0) < 0$, then $G(x) < 0$ for all the $x$ between 0 and 1. This induces $x^* = 0$ is the unique stable equilibrium for the dynamical system.

But $G(0) = ef(0) - r$. If $R_0 \leq \frac{1}{1-r}$ is valid, then $G(0) = e(1 - \frac{1}{R_0}) - r < 0$ is valid for any $e$ between 0 and 1. This completes the proof for $(i)$.

For $(ii)$:

for $R_0 \leq (\frac{1}{1-\sqrt{r}})^2$, we have that $G(1)$ is smaller than zero for any $e$. For $R_0 > \frac{1}{1-r}$, the sign of $G(0)$ depends on $e$: if $e \leq \frac{r}{1-\frac{1}{R_0}}$, it is negative, which results in all unvaccination eventually. Otherwise, it is positive. In this case, there is a unique equilibrium $x^*$ lying between 0 and 1 since $G(x)$ is continues and monotonic [2]. In addition to this, the derivative of $G(x)$ at $x^*$ is negative since $f(x)$ is decreasing. Therefore, there is a unique internal stable equilibrium $x^*$. By the $f(x)$ we adopt, we have $x^* = \frac{1-\frac{1}{R_0(1-\frac{r}{e})}}{e}$.

This completes the proof of $(ii)$.

For $(iii)$:

if $e \leq \frac{r}{1-\frac{1}{R_0}}$, we have $G(0) < 0$. In analogy to the proof in $(i)$, we have $x^* = 0$ is the unique stable equilibrium.

If $\frac{r}{1-\frac{1}{R_0}} < e \leq e_1^*$, then $G(0) > 0$ while $G(1) < 0$. In analogy to $(ii)$, there is a unique internal stable equilibrium $x^*$;

If $e_1^* < e \leq e_2^*$, we have $G(1) > 0$, since $G(x)$ is decreasing, we have $G(x)$ is positive for all the $x$ lying between zero and one. Thus, all vaccination becomes the unique stable equilibrium this time.

If $e_2^* < e$, then $G(0) > 0$ while $G(1) < 0$, In analogy to $(ii)$, there is a unique internal stable equilibrium $x^*$.

This completes the proof.

# References

1. Hofbauer J, Sigmund K (1998) Evolutionary Games and Population Dynamics. Cambridge: Cambridge University Press.

2. Rudin W (1986) Principles of mathematical analysis. McGraw-Hill.
